# Intraarterial route increases the risk of cerebral lesions after mesenchymal cell administration in animal model of ischemia

**DOI:** 10.1038/srep40758

**Published:** 2017-01-16

**Authors:** Bárbara Argibay, Jesse Trekker, Uwe Himmelreich, Andrés Beiras, Antonio Topete, Pablo Taboada, María Pérez-Mato, Alba Vieites-Prado, Ramón Iglesias-Rey, José Rivas, Anna M. Planas, Tomás Sobrino, José Castillo, Francisco Campos

**Affiliations:** 1 Clinical Neurosciences Research Laboratory, Clinical University Hospital, Health Research Institute of Santiago de Compostela (IDIS), Universidade de Santiago de Compostela, Santiago de Compostela, Spain; 2 IMEC, Department of Life Science Technology, Leuven 3001, Belgium; 3 Biomedical MRI, Department of Imaging and Pathology, KU Leuven, Leuven 3000, Belgium; 4 Department of Morphological Sciences, Universidade de Santiago de Compostela, Santiago de Compostela, Spain; 5 Grupo de Física de Coloides y Polímeros, Departamento de Física de la Materia Condensada, Universidade de Santiago de Compostela, Santiago de Compostela, Spain; 6 Departamento de Fisiología, Centro Universitario de Ciencias de la Salud, Universidad de Guadalajara, Guadalajara 44340, México; 7 Applied Physics Department, Campus Vida, Universidade de Santiago de Compostela, Santiago de Compostela, Spain; 8 Department of Brain Ischemia and Neurodegeneration, Institut d’ Investigacions Biomèdiques de Barcelona (IIBB), Consejo Superior de Investigaciones Científicas (CSIC), Barcelona, Spain; 9 August Pi i Sunyer Biomedical Research Institute (IDIBAPS), Barcelona, Spain

## Abstract

Mesenchymal stem cells (MSCs) are a promising clinical therapy for ischemic stroke. However, critical parameters, such as the most effective administration route, remain unclear. Intravenous (i.v.) and intraarterial (i.a.) delivery routes have yielded varied outcomes across studies, potentially due to the unknown MSCs distribution. We investigated whether MSCs reached the brain following i.a. or i.v. administration after transient cerebral ischemia in rats, and evaluated the therapeutic effects of both routes. MSCs were labeled with dextran-coated superparamagnetic nanoparticles for magnetic resonance imaging (MRI) cell tracking, transmission electron microscopy and immunohistological analysis. MSCs were found in the brain following i.a. but not i.v. administration. However, the i.a. route increased the risk of cerebral lesions and did not improve functional recovery. The i.v. delivery is safe but MCS do not reach the brain tissue, implying that treatment benefits observed for this route are not attributable to brain MCS engrafting after stroke.

Ischemic stroke, caused by interruption of the blood supply to the brain, is one of the most important causes of morbidity and mortality worldwide. Currently, the control of systemic parameters, such as body temperature, blood pressure, and glycemia, has considerably improved the outcome of stroke patients. However, in the absence of protective therapy, an early artery reperfusion, i.e. mechanical or enzymatic thrombolysis, remains the primary goal of treatment for acute ischemic stroke^1^,^2^.

Cell based therapies have emerged as a promising tool for the treatment of both acute and delayed phases of stroke. In this regard, mesenchymal stem cells (MSCs) are one of the best candidates for stem cell therapy of ischemic stroke owing to their multipotentiality, ability to release growth factors, and immunomodulatory capacities^3^. Thus, this transdifferentiation can produce cells with a neural lineage^4^,^5^,^6^,^7^, induce neurogenesis^8^,^9^,^10^, angiogenesis^8^,^9^,^10^ and synaptogenesis^11^, and activate endogenous restorative processes through production of cytokines and trophic factors^8^,^12^,^13^,^14^. Moreover, the regulation of cerebral blood flow (CBF), the blood brain barrier (BBB)^12^, and other neuroprotective mechanisms, such as the reduction of apoptosis, inflammation, demyelination, and increased astrocyte survival^8^,^9^,^15^,^16^, have been involved as beneficial mechanisms of MSCs after of stroke^3^.

Functional recovery in animal models of focal cerebral ischemia has been observed when MSCs were injected intravenously (i.v.) or intraarterially (i.a.)^17^,^18^,^19^,^20^, however, there is not agreement yet about the optimal administration route. Intravenous injections are minimally invasive, and cell tracking studies following that route have shown that most administered cells remain trapped in the lungs, liver, and spleen^21^, indicating that a reduced number of cells reach the brain^22^. Intraarterial administration is a promising strategy to direct the majority of injected cells to the brain^23^, but the fate of injected cells following this route remains unknown due to high variation in the reported results. Indeed, recent studies have shown that approximately 21% of the cells delivered via i.a. carotid injection were observed in the ipsilateral hemisphere^24^. Conversely, other studies have reported that 24 h after injection, 95% of the delivered cells were found in the spleen^25^. Additional studies have indicated that i.a. carotid cell administration is a safe delivery strategy that can overcome limitations of i.v. administration, since it represents a more direct route; however, new findings have associated a higher mortality to i.a. administration compared to the i.v. route^21^. Despite of the discrepancies about the best route for cell administration, it has also not been well established if the therapeutic effect described for MCSs after i.a. and i.v. administration requires the diffusion of cells through the BBB and the engraftment in the cerebral parenchyma tissue.

Therefore, an *in vivo* analysis of the cellular fate and biodistribution of both administration routes is an important and necessary step towards the further development of minimally invasive stem cell therapy for central nervous system diseases, including stroke. To this end, the objective of this study was to perform an analysis of cell tagging by magnetic resonance imaging (MRI) contrast agents (CAs) and subsequent MRI analysis to address this challenge^26^.

In this study, first, we synthesized dextran-coated superparamagnetic nanoparticles (D-MNPs), validated their use as CAs for cell tracking in MRI, and evaluated the cellular viability of MSCs after labeling, including their detection by MRI. Secondly, the optimal route and cell dosage were evaluated for i.a. administration. Third, cellular biodistribution patterns following i.a. and i.v. administration were investigated. Finally, the therapeutic effects of MSCs administered through either route were compared in an animal model of ischemic stroke.

## Results

### Synthesis and characterization of D-MNPs

**Figure 1:**
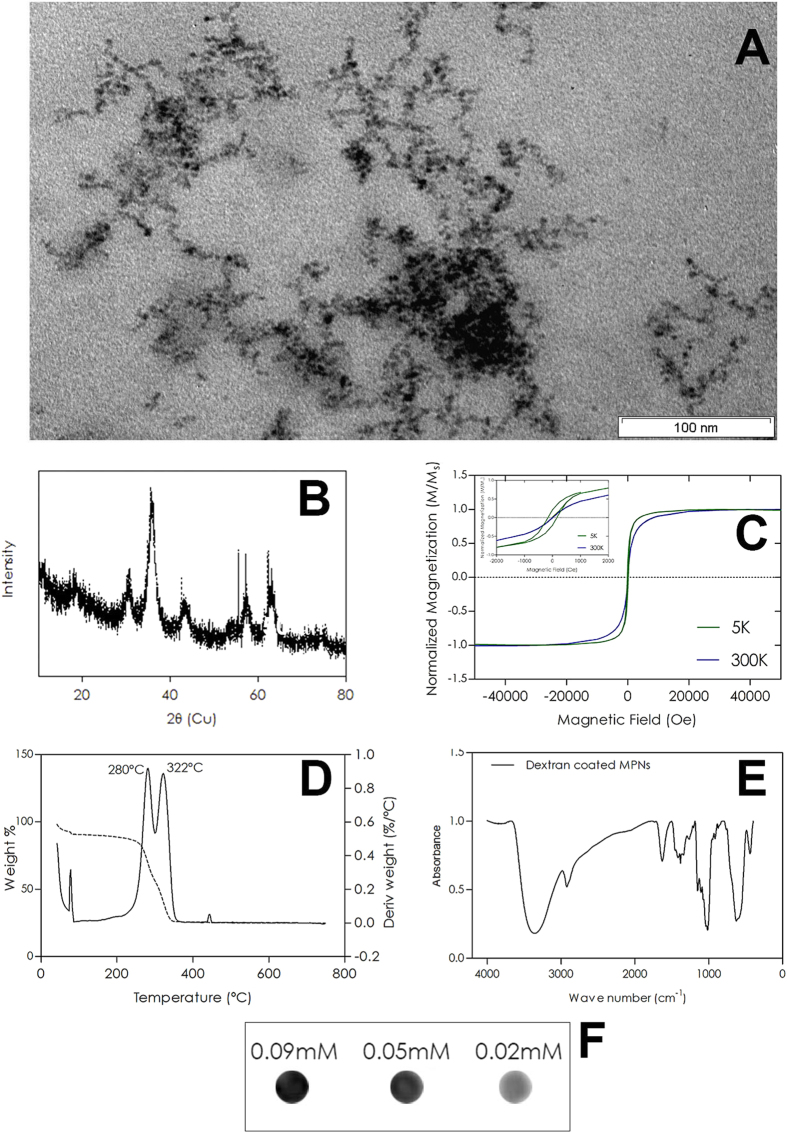
Characterization of D-MNPs. (**A**) TEM micrograph showing the morphology and size distribution of D-MNP cores; (**B**) The XRD spectrum of D-MNP cores synthesized through chemical co-precipitation shows the characteristic peaks of magnitude at 2θ positions of ca. 30.2°, 35.6°, 43.2°, 57.1°; and 62.7°; (**C**) Magnetization curve of D-MNPs demonstrates superparamagnetic behavior at room temperature; (**D**) TGA spectrum of D-MNPs and (**E**) FTIR spectrum of D-MNPs provide evidence for the presence of dextran in the D-MNP formulation; (**F**) T_2_-weighted MRI of 0.09 mM, 0.05 mM, and 0.02 mM D-MNPs. Iron concentrations demonstrate the suitability of D-MNPs as contrast agents (CA) for MRI analysis.

D-MNPs were synthesized in the presence of dextran following the chemical co-precipitation method described in the Methods section. Transmission electron microscopy (TEM) micrographs (Fig. 1A) showed a mean core size of 3.7 ± 0.8 nm. The core crystal structure determined by X-ray diffraction (XRD) (Fig. 1B) showed peaks at 2θ positions of ca. 30.2°, 35.6°, 43.2°, 57.1°, and 62.7°, corresponding to the (220), (311), (400), (511), and (440) planes of magnetite, respectively, with a lattice parameter of 8.33 ± 0.02 Å and a crystallite size of 4.8 ± 0.5 nm derived from the Scherrer equation. Vibrating sample magnetometer (VSM) measurements showed that cores exhibited superparamagnetic behavior at room temperature (Fig. 1C). The hydrodynamic size of D-MNPs measured by dynamic light scattering (DLS) was 94 ± 3 nm with Z-potential values of ca. −11 ± 3 mV. The presence of the polymeric coating was confirmed by thermal gravimetric analysis (TGA) and fourier-transform infrared (FTIR). The TGA spectrum (Fig. 1D) showed two peaks, corresponding to mass losses at approximately 280 °C and 322 °C, in the range of dextran decomposition temperature (150–380 °C)^27^, demonstrating that about 66 wt% of the D-MNPs can be attributed to dextran. The FTIR spectrum (Fig. 1E) exhibited several polysaccharide-characteristic absorption bands at 3351 cm^−1^ due to O-H stretching; vibrational modes ν (C-H) and δ (C-H) corresponding to 2906 cm^−1^, 1420 cm^−1^, and 1380 cm^−1^; water molecular bending assigned to 1620 cm^−1^, 1145 cm^−1^; and 1010 cm^−1^ peaks due to C-O vibrations; and small peaks at 908 cm^−1^, 877 cm^−1^, and 746 cm^−1^, corresponding to α-glucopyranose ring deformation modes. There were also two absorption bands at 578 cm^−1^ and 430 cm^−1^, corresponding to Fe-O vibration modes^28^,^29^. MRI contrast in T_2_-weighted images at different Fe concentrations (0.09 mmol/L; 0.05 mmol/L, and 0.02 mmol/L) of D-MNPs were measured (Fig. 1F). A linear relationship was found between R_2_(R_2_ = 1/T_2_) and MNP concentration, resulting in a transverse relaxivity (r_2_) of 701 ± 16 L mmol^−1^ s^−1^.

### Characterization of mesenchymal stem cells labeling with D-MNPs

**Figure 2:**
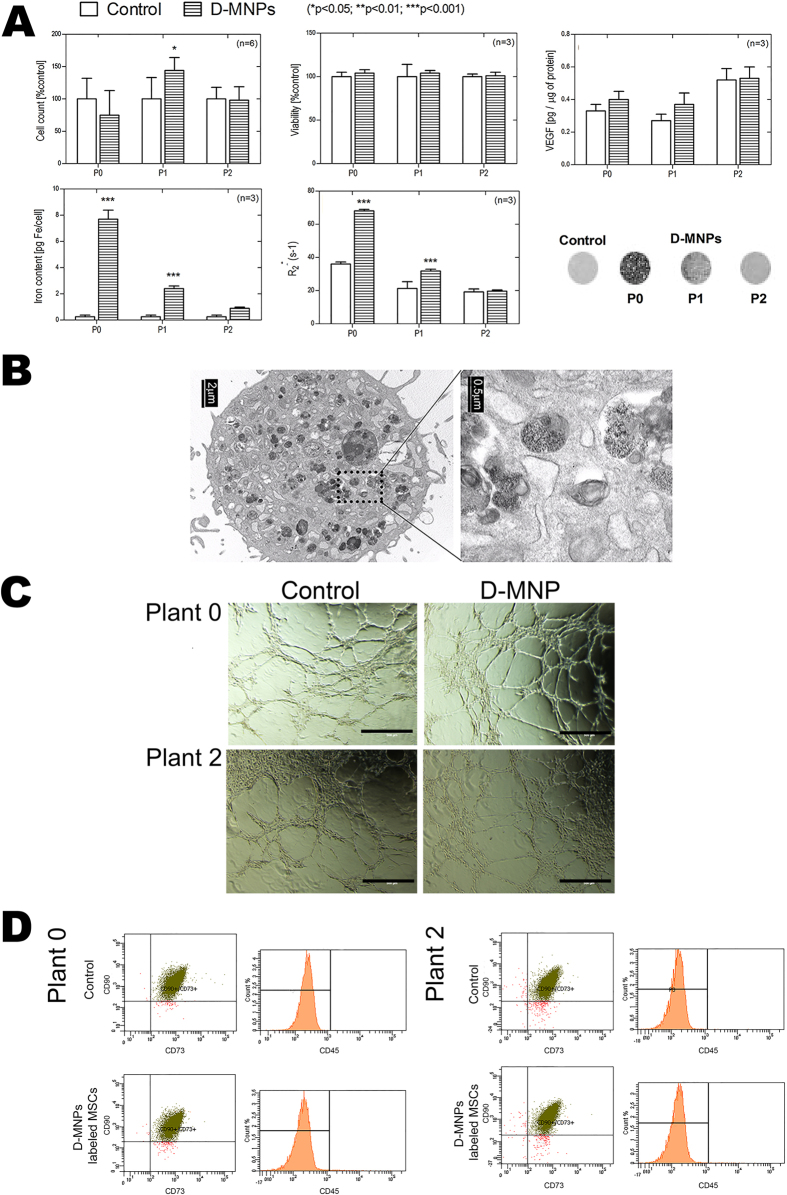
Time elapsed study of MSCs labeled with D-MNPs. (**A**) Cell count after labeling at P0, P1, and P2 compared to the control. Viability after labeling at P0, P1, and P2 compared to the control as determined by LDH assay. VEGF secretion of D-MNPs and the control at P0, P1, and P2. Iron content per cell at P0, P1, and P2, as determined by ICP. Relaxation times (R2*) of labeled and non-labeled cells at P0, P1, and P2. T2*-maps of control and D-MNP labeled cells at P0, P1, and P2. (**B**) TEM of a MSC labeled with D-MNPs appearing as dispersed black regions within the cellular cytoplasm. Magnification in one region indicates evidence of D-MNP encapsulation in endosomes. (**C**) Matrigel-angiogenic assay in Control and D-MNP labeled MSCs at P0 and P2. (**D**) Flow cytometry analysis of Control and D-MNP-labeled MSCs at P0 and P2 for CD90/CD73/CD45 analysis. P0 (12 h), P1 (3 days) and P2 (5 days) after D-MNP labeling.

The analysis of the cell properties showed that D-MNP labeling did not reduce the proliferation rate of MSCs with respect to the control when evaluated as cell counts at P0 (12 h), P1 (2 days), and P2 (5 days) after labeling (Fig. 2A). No significant differences were observed between D-MNPs and the control in terms of cell viability determined by lactate dehydrogenase (LDH) assay (Fig. 2A). Vascular endothelial growth factor (VEGF) release was not affected by D-MNP internalization with respect to control cells (Fig. 2A) at the different time-points analyzed (P0, P1, and P2). The amount of internalized iron oxide nanoparticles was determined in labeled cells by inductive coupled plasma optical emission spectroscopy (ICP-OES) (Fig. 2A). Internalized iron at P0 was 7.7 ± 0.7 pg Fe/cell, decreasing to 2.4 ± 0.2 pg Fe/cell at P1, and 0.90 ± 0.09 pg Fe/cell at P2. MRI analysis of labeled cells and non-labeled cells was also performed. T_2_*-maps were calculated from T_2_*-weighted images. R_2_* relaxivities of 500 cells/μL in agar were obtained for each condition (Fig. 2A). At P0, the R_2_* values were 36.0 ± 1.2 s^−1^ and 68.0 ± 0.9 s^−1^ for the control and D-MNPs, respectively; at P1, these values were 21 ± 4 s^−1^ and 31.8 ± 1.1 s^−1^ for the control and D-MNPs, respectively; and at P2, these values were 19 ± 2 s^−1^ and 19.6 ± 0.8 s^−1^ for the control and D-MNPs, respectively, according to the decrease in internalized iron observed at the different timepoints. Transmission electron microscopy (TEM) images of labeled MSCs showed D-MNPs engulfed in cellular compartments and distributed along the cell (Fig. 2B). No D-MNPs were observed attached at the surface of the cells. In addition, the matrigel assay did not show differences in angiogenic capacity between control and D-MNP-labeled cells at P0 and P2 in terms of ring formation and distribution (Fig. 2C). Finally, flow cytometry analysis revealed no differences in the membrane phenotype markers of MSCs (CD90^+^, CD73^+^ and CD45^-^) between D-MNPs and non-labeled MSCs at P0 and P2 (Fig. 2D). No differences between cell passages 9 and 17 were observed regarding to VEGF release, ring formation or membrane phenotype markers. Moreover, analysis of labeled cells with increasing amounts of D-MNPs did not affect cellular size (size average in suspension 15 ± 2 μm).

### Intra-arterial cell tracking of D-MNPs MSCs

**Figure 3:**
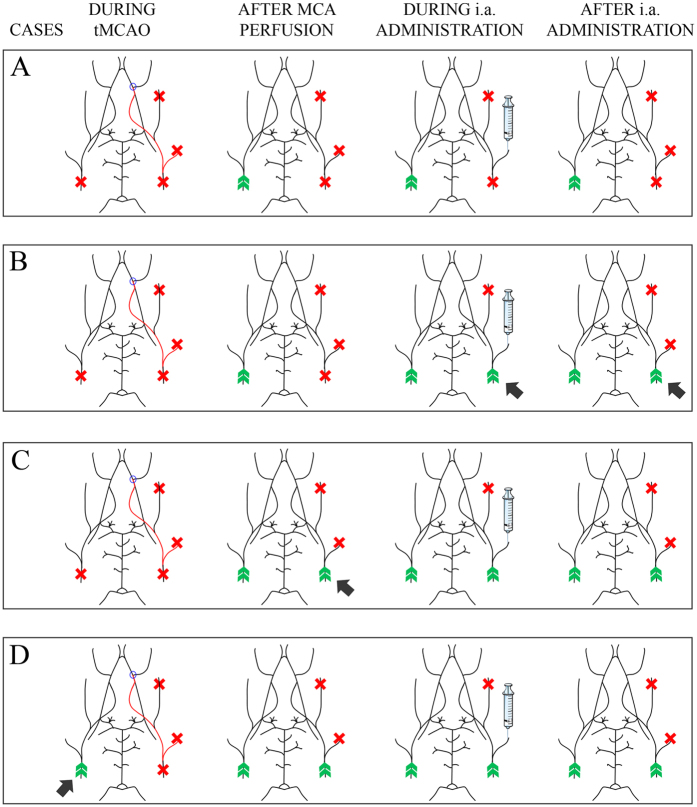
Several combinations of i.a. MSCs delivery were considered in this study to elucidate the most efficient and safe administration, here the schemes of different experimental procedures for tMCAo and i.a. cannulation for brain stem cell delivery. Black arrows denote differences between the indicated case and the previous case. **CASE A**: The Ip-CC and Con-CC were closed during tMCAo; immediately after reperfusion of the MCA, the con-CC was also reperfused. MSCs were administered after 4 h through the Ip-EC with the Ip-CC closed. After administration, the Ip-EC and Ip-CC remained closed. **CASE B**: The Ip-CC and Con-CC were closed during tMCAo; immediately after reperfusion of the MCA, the con-CC was perfused as well. MSCs were administered after 4 h through the Ip-EC with the Ip-CC opened. After administration, the Ip-EC remained closed, while the Ip-CC remained open. **CASE C**: The Ip-CC and Con-CC were closed during tMCAo; immediately following reperfusion of the MCA, both CCs were perfused. MSCs were administered after 4 h later through the Ip-EC with the Ip-CC opened. After administration, the Ip-EC remained closed and the Ip-CC remained open. **CASE D**: The Con-CC remained intact during all procedures. Immediately after reperfusion of the MCA, the con-CC was reperfused as well. MSCs were administered after 4 h through the Ip-EC with the Ip-CC opened. After administration, the Ip-EC remained closed, while the Ip-CC remained open. See Supplemental Figure S1 for arterial nomenclature. N = 8 per experimental case. The figure was produced, in part, by using Servier Medical Art, (www.servier.com/Powerpoint-image-bank).

To optimize i.a. cell delivery, previously we tested various combinations of surgical procedures in an animal model of cerebral ischemia as it is detailed in Fig. 3. In all the experimental conditions MSC administration (1 × 10^6^ MNP labeled MSCs suspended in 300 μL) was carried out 4 hours after transient middle cerebral artery occlusion (tMCAo).

In administration protocol A (Fig. 3A), a high variation was observed in cerebral MSC detection. MSC administration following procedure A resulted in brain engraftment in only 3 out of 8 animals, and no associations with mortality were found. In this procedure, the ipsilateral common carotid (Ip-CC) remained closed from the beginning of tMCAo that could limit the entrance of labeled cells after i.a. administration.

To overcome this effect, in protocol B (Fig. 3B), cerebral blood flow in the Ip-CC was reestablished at the time of administration. In this experimental condition, the Ip-CC was opened during the injection, ensuring high engraftment efficiency, but most of the animals died (6 out of 8 rats died within 24 h of cell delivery). This high mortality could be due to secondary intracerebral hemorrhage since the Ip-CC was closed for 4 h prior to injection, which could induce vessel damage.

In protocol C (Fig. 3C), the Ip-CC was opened after tMCAo to prevent the possible vessel damage caused by prolonged occlusion. Under these conditions, all the animals showed hypointense signals in MRI, but exhibited difficulties in breathing when they awoke from anesthesia, and 4 out of 8 rats died within the next 5 days. Coronal MR T_2_*-weighted images showed dispersed cells along the forebrain and also in the cerebellum suggesting cell trafficking through the posterior communicating artery, which connects the vertebro-basilar system with the carotid system (diagram of cerebrovascular anatomy is represented in the Supplementary Figure S1).

**Figure 4:**
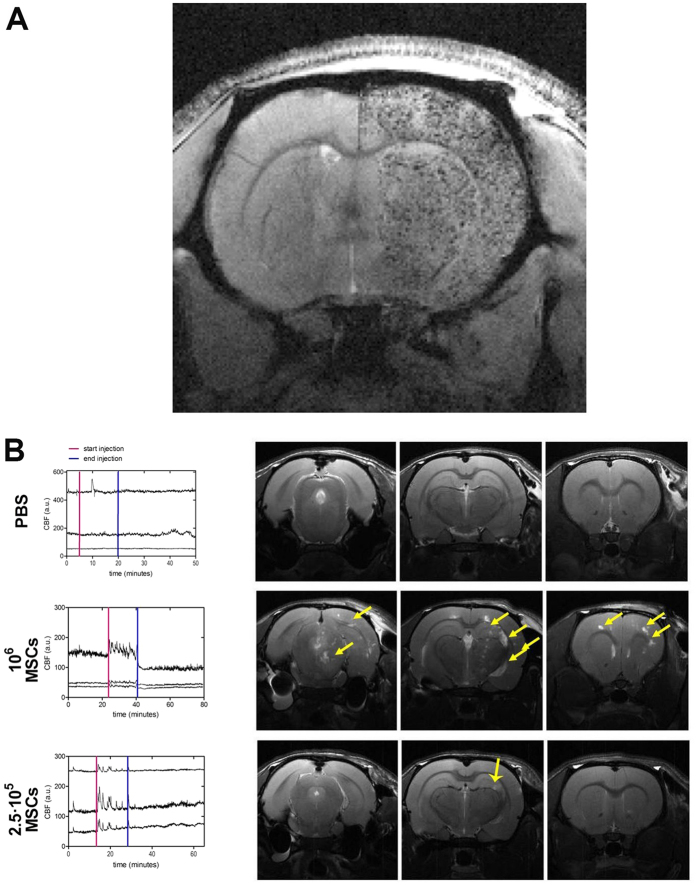
(**A**) First echo of MR T_2_* weighted image of one brain slice of a *Wistar* rat 8 h after the onset of cerebral ischemia, and 4 h after i.a. administration of 1 × 10^6^ MSCs labeled with D-MNPs in case D. Labeled cells can be observed as black punctate patterns in the right hemisphere of the animal. (**B**) Cerebral blood flow in three regions of the middle cerebral artery determined in healthy animals by 3 Doppler probes before, during, and after administration of PBS, 0.25 × 10^6^ non-labeled MSCs, and 1 × 10^6^ non-labeled MSCs; MR T_2_-weighted images of several brain slices 24 h after cell delivery. Multifocal ischemia is observed all along the brain after administration of 1 × 10^6^ MSCs (indicated with yellow arrows). After the administration of 0.25 × 10^6^ MSCs, an isolated micro-ischemia was observed in one animal (indicated with a yellow arrow). No edema (middle line shift) was observed for all conditions studied. N = 6 per experimental group.

After occlusion of both common carotids (Fig. 3A–C), blood flow to the forebrain is provided by the vertebro-basilar system through the posterior communicating arteries, which suffer vasodilatation. After cell injection and under these conditions, the regional localization of cells along the brain and cerebellum depends on the CBF distribution in the circle of Willis. To test whether blood supply through the common carotid (Con-CC) could prevent cell migration towards the cerebellum, in protocol D (Fig. 3D) this artery was not closed during tMCAo. The results showed uniform cerebral cell distribution and no mortality. Typical brain engraftment of D-MNP labeled MSCs could be observed in T_2_*-weighted images (Fig. 4A) after this delivery route. All these results are summarized in the Supplemental Table S1.

To determine the safety of different cell doses in i.a. administration procedure D, two cell doses, 0.25 × 10^6^ and 1 × 10^6^ non-labeled MSCs in PBS (used as the cell vehicle) were injected in healthy animals and were compared to vehicle administration. CBF was monitored during delivery, and brain was evaluated 24 h after administration by MR T_2_-weighted images. MR images showed absence of lesions in the cerebral tissue of rats receiving PBS (Fig. 4B). In contrast, administration of 1 × 10^6^ MSCs induced multifocal lesions in all brain slices (Fig. 4B), and injection of 0.25 × 10^6^ MSCs produced only one isolated lesion in one of the animals without evidence of brain edema. Multifocal lesions caused by the 1 × 10^6^ dose of MSCs were also associated with a reduction of CBF determined by laser Doppler flowmetry. Accordingly, we limited the cell delivery in this study to 0.25 × 10^6^ MSCs as a safe dose for i.a. administration.

**Figure 5:**
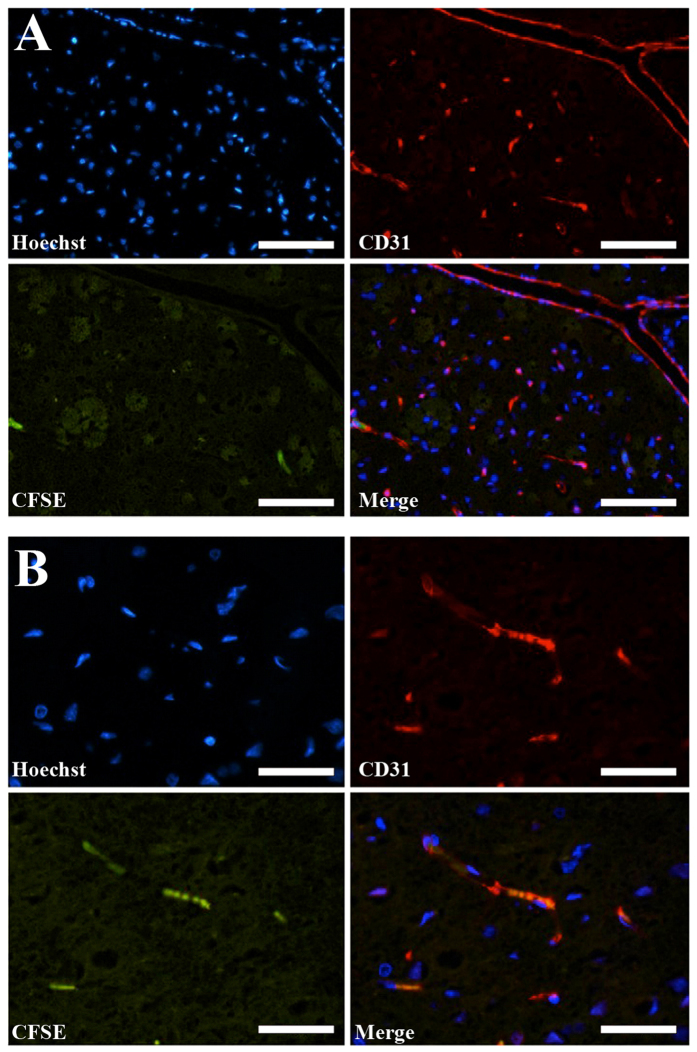
Histological cell localization after intraarterial (i.a.) administration of CFSE labeled MSCS. Positive staining of injected MSCs can be seen in the small diameter vessels (**A** and **B**) but not in big vessels (**A**). CFSE is co-localized with CD31 and Hoechst. (Scale bar 100 μm).

**Figure 6:**
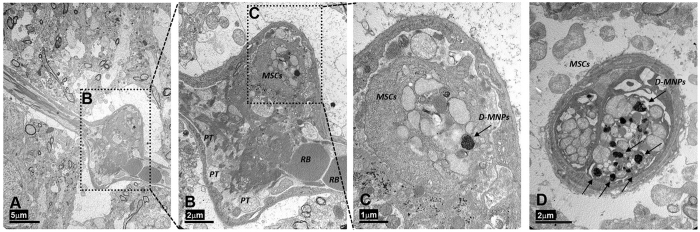
Electron transmission micrograph of rat brain cortex 4 h after intra-arterial (i.a.) delivery of D-MNP-labeled MSCs. (**A**) Dilated brain vessel surrounded by neuropil (Scale bar 5 μm). (**B**) Magnification of vessel dilation in (**A**). Red blood (RB) corpuscles are on the right, and platelets(PTs) and MSCs can be observed inside the vessel (Scale bar 2 μm). (**C**) Magnification of the upper vessel expansion in (**B**). One well-defined MSC is identified by the encapsulated D-MNPs (black punctate encapsulated pattern). (Scale bar 1 μm). (**D**) Longitudinal section of a brain vessel where two D-MNPs labeled MSCs can be observed. Encapsulated D-MNPs are noted by dark punctuate patterns (Scale bar 2 μm).

**Figure 7:**
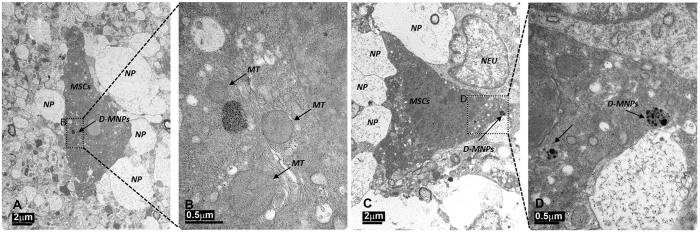
Transmission electron micrograph of rat brain cortex 4 h after intra-arterial (i.a.) delivery of D-MNP-labeled MSCs. (**A**) One single MSC labeled with D-MNPs was found in the brain parenchyma surrounded by neuropil (NP) (Scale bar 2 μm). (**B**) Magnification of the region selected in **A**, where D-MNPs are observed as black punctate regions compartmentalized within a membrane. Three mitochondria (MT) can be noted as well (Scale bar 0.5 μm). (**C**) One single MSC labeled with D-MNPs was found in the brain parenchyma surrounded by neuropil (NP) and by a neuron (NEU) (Scale bar 2 μm). (**D**) Magnification of the region selected in **C**, where D-MNPs can be distinguished as black punctate regions compartmentalized within the membrane (Scale bar 0.5 μm).

To further study the distribution of MSCs and to determine the possible cause of multifocal lesions observed previously, healthy animals were treated (procedure D) with 1 × 10^6^ MSCs labeled with D-MNPs and CFSE (Cell Proliferation Kit is used for *in vivo* labeling of cells) for MRI and histology detection, respectively. T_2_*-weighted images showed D-MNPs labeled MSCs in the brain of all animals. Immunohistological analysis revealed positive staining for nuclei (Hoechst), MSCs (CFSE), and vessels (CD31) mainly in the ipsilateral hemisphere along all cerebral sections. CFSE positive MSCs were mainly observed in small vessels (Fig. 5A,B) but not in wide vessels, and we found no evidence of cells extravasated to the brain parenchyma. Immunohistological analysis of lungs and heart did not reveal CFSE positive MSCs. In line with these results, TEM analysis showed MSCs in small vessels (Fig. 6), however, labeled MSCs were also detected in the brain parenchyma (Fig. 7). MSCs were identified readily due to their contrasting cytoplasmic color compared to the surroundings and scattered dark endosomes within the cellular membrane. Magnification in one region showed a dark, punctate pattern inside the endosome membrane cristae, with similar distribution, size, and morphology to those previously observed *in vitro* (Fig. 2B). Adjacent to the endosomes, mitochondria were also identified by their cristae, demonstrating the viability of MSCs after injection.

### Intravenous cell tracking of MSCs

Cell tracking after i.v. administration of 1 × 10^6^ MSCs labeled with D-MNPs and CFSE showed no cerebral engraftment, noted as hypointensities in MR T_2_*-weighted images, in any of the rats. This *in vivo* result was confirmed by histological analysis. No positive CFSE staining for MSCs was found in the brain and heart by using fluorescence optical microscopy, whereas positive CFSE-labeled cells were found to be distributed along the lungs (Supplemental Figure S2).

### Neurorecovery study

**Figure 8:**
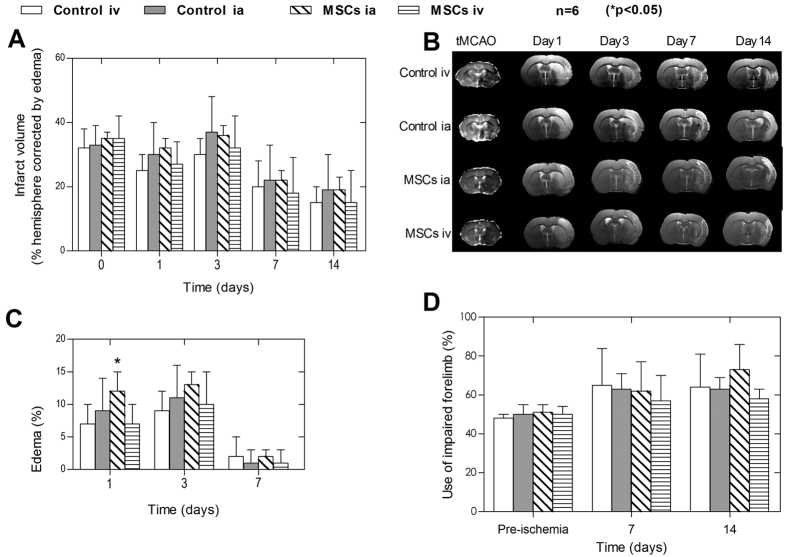
Neurorecovery after i.a. and i.v. administration of MSCs. (**A**) Infarct volumes (% hemisphere and corrected by edema) measured from MR apparent diffusion coefficient (ADC) (tMCAo) and T_2_-map (days 1 to 14). (**B**) Representative follow-up MRI of each study group (ADC maps images for tMCAo and T_2_ weight images for 1, 3, 7 and 14 days). (**C**) Edema volume (% increase in ipsilateral hemispheric volume with respect to contralateral hemispheric volume). (**D**) Use of impaired forelimb (%) as determined by cylinder tests (N = 6; *P < 0.05).

Once the optimal injection protocol and MSC dosage for safe i.a. administration and the distribution of MSCs after i.v. and i.a. delivery in rats were established, we aimed to evaluate the neurorecovery effect produced by i.a. and i.v. administration of MSCs after cerebral ischemia. D-MNP labeled MSCs were detected readily in brain by MR T_2_*-weighted images at 4 h, 24 h, and 3 days after i.a. injection only (Supplemental Figure S3). Infarct volume analysis revealed that both administration route did not reduce the infarct volume within 14 days after tMCAo, compared to the groups receiving the vehicle. In fact, lesion size significantly increased after i.a. delivery of PBS or MSCs with respect to i.v. treatments (Fig. 8A,B). The administration of MSCs, either i.a. or i.v., did not reduce edema volume in the acute phase of cerebral ischemia. Moreover, a significant increase in edema volume was observed 24 h after i.a. administration (*P < 0.05) (Fig. 8C). Functional recovery after MSC delivery was also evaluated by the cylinder test, and no significant differences were observed between groups (Fig. 8D). Nonetheless, the group of rats receiving i.v. MSCs showed a reduction of the use of the impaired forelimb, but the difference did not reach statistical significance compared to the i.v. control group.

**Figure 9:**
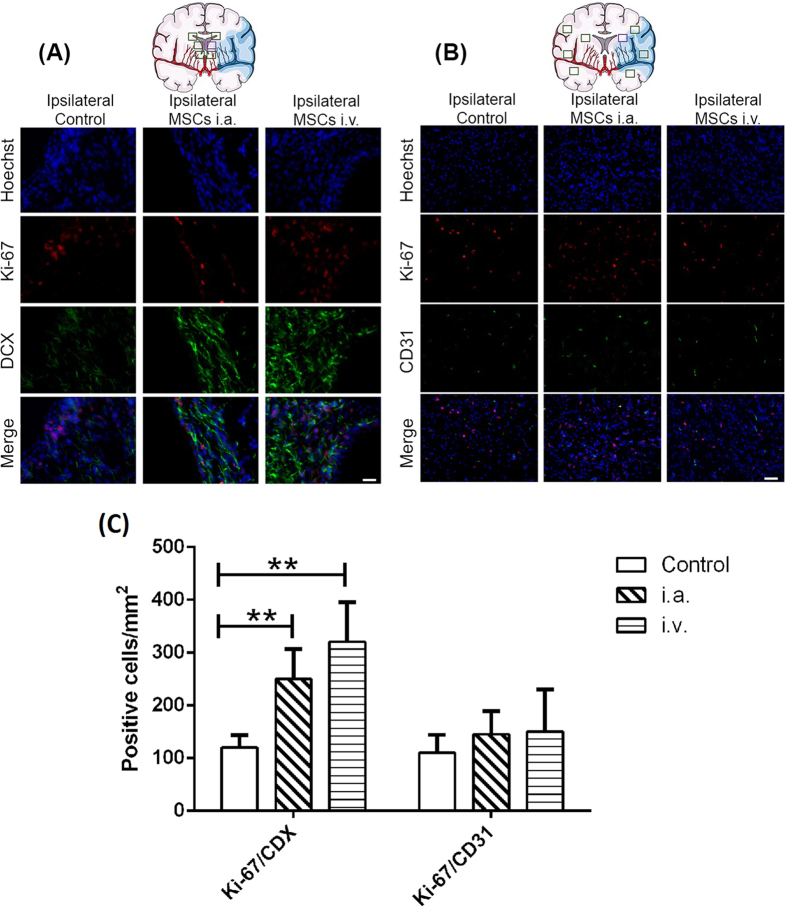
(**A**) Immunohistofluorescence of subventricular zones to reveal neurogenesis using DCX and Ki-67. Representative images of different groups are shown corresponding to the blue rectangle in the scheme. (**B**) Immunohistofluorescence of peri-infarct region to evaluate the angiogenesis by using CD31 and Ki-67. Representative images of the different groups are shown corresponding to the red rectangle in the scheme. Scale bar represents 50 μm. (**C**) Analyses of positive cells for Ki-67 and CDX or CD31 makers in different brain regions of control and treated (i.a. and i.v.) animals. Data are shown as mean ± SEM. **P < 0.01; using two-way ANOVA followed by the post-hoc Bonferroni test, (n = 3, per group). The figure was produced, in part, by using Servier Medical Art, (www.servier.com/Powerpoint-image-bank).

The plasma concentrations of VEGF at different time points were below the limit of detection of the ELISA assay used in this study. The presence of neural progenitors in the subventricular region was examined with doublecortin (DCX) and Ki-67 immunostaining (Fig. 9A). Co-localization of DCX and Ki-67 was higher for MSC administered groups compared to controls, particularly for DCX staining, a marker of neuronal precursor cells and immature neurons (Fig. 9A and C). Infarct and peri-infarct regions were examined to evaluate angiogenesis through co-localization of CD31 and Ki-67, but no differences in marker staining were observed between groups (Fig. 9B and C).

## Discussion

MSCs have received special attention in recent years as a promising therapeutic candidate for stroke due to their potential multi-mechanistic effects on the damaged brain^30^. However, parameters such as the therapeutic window and cell dosage or administration route are still under discussion^31^,^32^. For a better understanding of how stem cell therapies can succeed, non-invasive monitoring of cells is required, and MRI is one of the most appropriate tools for this use because it provides excellent soft tissue contrast and high resolution^33^,^34^,^35^. MRI can provide anatomical and pathological information and, by tagging cells with MRI CAs such as superparamagnetic nanoparticles, MRI can establish the relationship between cellular biodistribution and outcome after stroke.

Indeed, the fate and biodistribution of injected MSCs after stroke is a critical aspect for therapeutic efficacy; therefore, the administration route is a decisive parameter. The most common routes of MSC transplantation are i.v. or i.a. infusion, or direct intra-tissue injection^36^. Although intraparenchymal (i.p.) administration provides direct cell delivery to the lesion, it results in poor lesion distribution and is highly invasive due to the necessary craniotomy^23^. Many studies have explored i.a. and i.v. administration in animal models of ischemic stroke with different degrees of success in terms of outcome^37^,^38^,^39^ and brain engraftment^23^,^40^,^41^,^42^, but only a few have established a relationship between them^43^,^44^,^45^. In this study, we administered MSCs labeled with D-MNPs to address the relationship between cell allocation and animal recovery after stroke.

Superparamagnetic iron oxide nanoparticles are the most frequently used CAs for cell tracking owing to the high hypointense MR signal in T_2_ and T_2_*-weighted images at low nanoparticle concentrations^46^. Several methods have been described for synthesizing these particles^47^. However, chemical co-precipitation is the easiest, most economical, and efficient in terms of producing a large amount of nanoparticles^48^,^49^. Our synthesis resulted in well-coated, size-uniformed, slightly negatively charged and homogeneously dispersed nanoparticle suspensions in aqueous solutions, similar to other nanoparticles previously used^28^,^29^,^50^,^51^,^52^. D-MNPs exhibited superparamagnetic behavior at room temperature, reflecting their suitability as a general MRI CA.

Although these nanoparticles can be used directly as a CA for MRI^53^,^54^,^55^, *in vivo* cell tracking requires nanoparticle internalization into the cells, which is a challenging procedure, as both are negatively charged^56^. To overcome this pitfall, Poly-L-lysine (PLL) (see methods for cell labeling with D-MNPs) was used because it has been widely accepted for nanoparticle cell tagging based on its low toxicity profile and high iron content per cell at low incubation concentrations^57^,^58^,^59^,^60^,^61^. Indeed, labeling was highly efficient, as particles were found inside the cells when observed by TEM, reducing the risk of unspecific contrast and possible MRI misinterpretation^59^,^62^. Quantitatively, the internalization of D-MNPs at P0 is in accordance with previous studies which have demonstrated the requirement of PLL for efficient cell labeling with commercial and in-house-synthesized dextran coated nanoparticles, obtaining comparable nanoparticle internalizations, up to 20 pg/cell^59^,^63^,^64^. However, since the administered cells must survive and interact with other cells in the host, an *in vitro* study of long-term detection and stem cell biology was necessary. Thus, labeled cells were seeded sequentially at P1 (2days) and P2 (5days), and their internalized iron and consequently T_2_*-MRI signals were found to be reduced. However, D-MNP-labeled MSCs were still detected by MRI immediately after labeling (P0) and 2 days later (P1). The MRI signal from labeled cells at P2 could not be distinguished from the control; the lower detectability limit for labeled cells has been reported previously to be 2–5 pg Fe/cell^61^,^65^. This dilution factor over time is related to the *in vitro* proliferation rate of MSCs (approximately two cell divisions, each lasting 24 h), which suggests that other cell types with therapeutic potential but different proliferation ratios could be labeled for longer or shorter periods of time.

Despite the indispensable MRI detection capacities of D-MNPs, cellular viability after labeling must be guaranteed for further effective therapy. One of the most relevant mechanisms of action of MSCs includes the release of VEGF, which promotes the development of fibroblasts, endothelial cells, and tissue progenitor cells, and performs tissue regeneration and repair^66^. In this study, labeled and non-labeled cells secreted equal levels of VEGF immediately after labeling and also 5 days after tagging, and MSCs maintained angiogenic capacity for tubular formation. Unchanged surface markers CD45^−^, CD90^+^, and CD73^+^ measured by flow cytometry^67^,^68^,^69^,^70^ after 5 days support the biocompatible nature of D-MNPs for MSC tagging.

The efficient labeling of MSCs *in vitro* led to the *in vivo* study of MSC biodistribution after delivery. Several studies have reported that the i.a. administration of stem cells is not a trivial issue, with high risk of secondary embolisms and high mortality^23^,^25^,^37^,^71^,^72^,^73^,^74^,^75^,^76^,^77^ as we have confirmed in our study. However, despite the risk of secondary embolisms, herein we have also observed that relatively small variation in carotid arteries occlusion, during tMCAo and i.a. cell administration has an enormous impact on cellular biodistribution and the survival of the animals. To our knowledge, this is the first work investigating the influence of the arterial cerebral system on cell biodistribution and also provides a tMCAo model for a safe an efficient i.a. cell delivery procedure, potentially useful for the administration of labelled MSCs or other types of cells. In brief, after tMCAo, the Ip-CC is perfused to prevent hemorrhagic transformation^78^, and the Con-CC is opened to provide sufficient blood supply to push the injected cells to the brain. Thus, labeled cells were observed in the brain of all animals and all survived after administration.

Intraarterial administration flow is another important parameter that must be considered to prevent the occurrence of micro-strokes in cell therapy. Indeed, previous studies performed in healthy animals^79^ have suggested that i.a. cell injections should be performed slowly, at a rate of 200 μL/min (1 mL) to reduce the risks of secondary embolisms. In our study, we have even observed that a lower rate of 20 μL/min (300 μL) cause secondary embolisms when the cell dose is higher than 0.25 × 10^6^ cells. Although experimental differences such as surgery procedure, surgery time, cell culture protocols, type of anesthesia used, animal blood pressure during cell administration or even animals strain (Wistar *vs* Sprague-Dawley) could explain the differences on optimal injection rate, both results evidence the risk of secondary embolisms during i.a. cell administration.

To elucidate the cause of cerebral lesions induced by i.a. cell administration and to determine whether the injected cells localized in vessels or migrated to the brain parenchyma, the cerebral tissue was analyzed by histology. CFSE positive cells were found in small diameter vessels suggesting vessel blockade that could explain why i.a. injection induced multifocal ischemia. However, tissue processing for immunofluorescence analysis could perturb cell localization since the animals must be perfused to eliminate the blood. On the contrary, TEM does not require animal perfusion and D-MNP-labeled MSCs were unequivocally detected because the D-MNPs were encapsulated in the cell endosomes. Brain cortex TEM revealed the presence of D-MNP-labeled MSCs occluding brain vessels, confirming the optical immunofluorescence observations and the origin of the lesions observed in the MR T_2_-weighted image analysis. In addition, TEM allowed detection of D-MNP-labeled MSCs in the brain parenchyma. Isolated MSCs migrated from vessels to the parenchyma, being in direct contact with the neuropil, where somas and cellular processes have been observed^80^.

Labeled cells were found in the brain parenchyma, evidence that after i.a. administration in a healthy rat, MSCs can cross the BBB. Other studies have assessed the capacity of MSCs to cross the BBB in healthy animals as well in ischemic stroke, Alzheimer disease, and epileptic animals models^81^; however, to our knowledge, this is the first work that directly demonstrates MSCs in the brain parenchyma in healthy rats.

In this study, the biodistribution of MSCs after i.v. administration was demonstrated to be definitively different from that after i.a. delivery. *In vivo* MRI combined with immunofluorescence analysis showed that after jugular administration (i.v.) of CFSE and D-MNP-labeled MSCs, MSCs were retained in the lungs. Although organs like liver, spleen or heart are potential organs where cells can be trapped after i.v. administration, several studies have described the lungs as the major obstacle for intravenous cell delivery; as a consequence, a reduced proportion of injected cells could reach the arterial circulation and thus the brain. Cell size and receptor-mediated adhesion or stem cell type appear to be crucial variables for pulmonary stem cell passage^22^,^82^.

More importantly, the finding that no cells were observed in the brain 24 h after administration confirms the non-engraftment of MSCs in the brain after i.v. administration, a salient point that has been discussed widely^23^,^41^,^43^,^44^,^45^.

In this study, we also examined the therapeutic effect after i.a. and i.v. administration, considering previous findings that (1) MSCs can be labeled with D-MNPs without side effects and (2) their biodistribution is dependent upon the route of administration. Our administration window was 4 h after the onset of ischemia, since several groups investigating the therapeutic window for stem cell administration in animal models of cerebral ischemia have suggested that early intervention could reduce lesion size and improve the functional outcome^44^,^83^.

Cells administered for the neurorecovery study were labeled with D-MNPs, and the animals were monitored for 14 days. Intraarterial administration of 0.25 × 10^6^ labeled MSCs resulted in detectable brain engraftment 3 days after delivery, whereas it was not possible to detect labeled cells in brains of animals injected i.v. with 1 × 10^6^ MSCs during the 14 days of evaluation.

Under our experimental conditions, no significant differences in infarct volume reduction and edema formation were observed for MSCs-treated and non-treated animals; in fact, i.a. delivery produced larger infarct volumes and edema formation than control or i.v. administration. A slight improvement in functional recovery was observed for i.v. administered cells, although this result did not achieve significance. It could be tentative to speculate that additional functional tests could contribute to find higher significant differences between the groups studied. However, among the different behavioral or neurological tests used for ischemic animal outcome evaluation, in our hands, cylinder test used represents one of most sensitive for functional evaluation of animal outcome, for this reason we have used this test as gold standard test for the cell therapy evaluation.

These results are in accordance with previous studies that have reported that stem cell therapies improved neurological function recovery without reduction in lesion volume^84^,^85^,^86^. Parameters such as the animal model, dosage, and therapeutic window have been shown to highly influence infarct volume reduction and functional recovery^39^,^43^,^83^,^84^,^87^.

Favorable effects on behavioral outcomes after an ischemic insult are normally associated with regenerative processes in the microenvironment, namely neurogenesis and angiogenesis^88^,^89^,^90^. Thus, in our study, we determined that treatment with MSCs significantly increases Ki-67 and DCX immunoreactivity in the ipsilateral subventricular zone, suggesting that MSC treatment enhances endogenous neurogenesis, but does not differ between i.a. and i.v. administration. Neurogenesis was reported previously by other authors following intrathecal and intraparenchymal administration of MSCs^91^,^92^. On the contrary, CD31 plus Ki-67 did not show an increase in angiogenesis in treated animals compared to the control 14 days following cerebral ischemia.

Previous studies have shown that human MSCs administered i.v. in rats after stroke promoted recovery through increases in endogenous levels of VEGF, VEGFR2, and basic-fibroblast growth factors^93^,^94^. In this study, we demonstrated that D-MNP-labeled MSCs secrete equal levels of VEGF compared to non-labeled cells *in vitro*, but MSC administration did not increase blood serum levels sufficiently to be detected in our ELISA assay.

Taken together, our findings indicate that i.v. administration of 1 × 10^6^ MSCs can improve functional outcome after cerebral ischemia to an extent, which is in agreement with several studies that have shown the remote immunomodulatory effects of these cells^95^,^96^. In this way, MSCs entrapped in the lung would affect immune system function systemically, supporting the hypothesis that beneficial MSC effects after stroke can be achieved in the absence of significant MSC recruitment to the infarct region, even overcoming the associated risks that i.a. administration represents^97^. In addition, MSCs after an experimental stroke have been found to secrete a wide number of neuroprotective and neurotrophic factors that promote repair and recovery through numerous pathways^97^. These secreted trophic factors would influence the neurogenesis observed 14 days after stroke, as it has already been reported^3^ however, brain engraftment does not seem to be a determinant, since neurogenesis has been observed following i.a. and i.v. delivery of MSCs.

Several limitations of this study have to be addressed. We have studied the cellular wellbeing of labeled cells for 5 days, but since these cells are aimed to survive and interact with the host cells, a longer-term study on their evolution would be necessary. On the other hand, we have chosen 4 hours for i.a and i.v administration after ischemia as the optimal therapeutic window for cell therapy based on previous studies^44^,^83^, however we cannot exclude the possibility that other time windows (shorter or longer than 4 hours), could induce benefic effect for i.a. administration. Although, the results reported to date about the optimal-time window for cell therapy are difficult to compare and still it is not a well-established timing for cell transplantation. Finally, in the neurorecovery study we have compared 0.25 × 10^6^ cells for i.a. administration *vs* 1 × 10^6^ cells for i.v. administration which could explain why i.v. route showed a greater effect. In this study we have used a dose of 0.25 × 10^6^ cells for i.a. administration because this was the safest dose for i.a. For i.v. administration, we have used 1 × 10^6^ because this is the most common dose for i.v. administration in rodents and because there are so many studies that have reported that doses lower than one million do not have effect on stroke improvement^39^,^43^. Therefore we decided to compare the highest and safest cell dose for i.a. *vs* the minimal and most common i.v. cell dose that could possibly be expected to have an effect.

In conclusion, MSCs were found in the brain following i.a. but not i.v. administration in ischemic rats. However, the i.a. route increased the risk of cerebral lesions and did not improve functional recovery. The i.v. delivery is safe but MCSs do not reach the brain tissue, implying that treatment benefits are not attributable to brain MCS engrafting after stroke.

## Materials and Methods

### Cell labeling with D-MNPs combined with Poly-L-Lysine

MSCs were labeled with D-MNPs following the protocol previously described^59^. See Supplementary Data for additional D-MNP synthesis and cell labeling characterization with D-MNPs.

### Experimental animals

All procedures involving the use of research animals were approved by the Research Committee of the University Clinical Hospital of Santiago de Compostela (Spain) and were performed according to the “Principles of Laboratory Animal Care” (NIH publication No. 86–23, revised 1985), as well as specific Spanish (RD 1201/2005 and RD 53/2013) and European Union (Directives 86/609/CEE, 2003/65/CE, 2010/63/EU) regulations.

Male Wistar rats (Harlan Laboratories, Barcelona, Spain) weighing 290 ± 10 g were kept in a controlled environment at 22 ± 1 °C and 60 ± 5% humidity, with 12:12 h light: darkness cycles and were fed *ad libitum* with standard diet pellets and tap water. All surgical procedures and MRI studies were conducted under sevoflurane (Abbott laboratories, IL, USA) anesthesia (3–4%) using a carrier 65:35 gas mixture of N_2_O:O_2_. All animals used in the study (included and excluded) are summarized in the Supplemental Figure S4.

### Transient ischemic stroke model

Transient focal ischemia (45 min) was induced by intraluminal occlusion of the left middle cerebral artery (tMCAo), following the method described elsewhere^98^. Briefly, under a surgical microscope, the ipsilateral common carotid (Ip-CC), ipsilateral external carotid (Ip-EC), and ipsilateral internal carotid (Ip-IC) arteries were exposed and dissected from connective tissue through a midline neck incision (Supplemental Figure S1). Ip-EC and pterygopalatine (PT) arteries were separated and tied with 6–0 silk sutures. In our model, common carotid (Con-CC) was also tied during surgery in order to obtain higher reproducible infarct volumes (see Intra-arterial administration protocol). Then, a silicon rubber-coated monofilament (403512PK5Re; Doccol Corporation, Sharon, MA, USA) was inserted through the Ip-EC into the CC artery and advanced into the IC to occlude the origin of the MCA. Cerebral blood flow was monitored with a Periflux 5000 laser-Doppler system (Perimed AB, Sweden) by placing the Doppler probe (model 411, Perimed AB, Sweden) in the parietal bone surface near the sagittal crest, under the temporal muscle. Once the artery was occluded, as determined by Doppler signal reduction, the anesthetized animals were moved carefully in less than 1 min from the surgical bench to a MR, to determine the basal ischemic lesion apparent diffusion coefficient (ADC) maps. In addition, angiography imaging was performed to confirm the artery remained occluded during the MR study. After basal MR analysis, animals were returned to the surgical bench for subsequent MCA reperfusion (45 min after occlusion). After surgery, animals were allowed to wake from anesthesia and to maintain a free moving gait until cell administration (4 h after reperfusion). Only animals with a CBF reduction higher than 60% in Doppler, MRI angiography validation of MCA occlusion, ADC hemispheric infarct volume ≥35 ± 10%, and complete reperfusion after MCA occlusion were included in the study.

### Intra-arterial administration of MSCs for *in vivo* cell tracking

The different experimental conditions tested for i.a. cell administration required testing the effect of contralateral common carotid (Con-CC) occlusion during tMCAo, and Ip-CC occlusion during and after MCAo, (see below in Fig. 3 and Supplemental Table S1). Vertebral arteries remained intact during all surgical procedures. Four hours after ischemia, animals were anesthetized and 1 × 10^6^ MNP labeled MSCs suspended in 300 μL of PBS were administered manually and carefully (perfusion rate: 20 μL/min) through the Ip-EC for *in vivo* cell tracking following the 4 different experimental conditions indicated in Fig. 3 (N = 8, each experimental condition). MR T_2_*-weighted images were acquired 4 h after administration to evaluate cellular engraftment in the brain.

For i.a. cell administration, a catheter (polythene tube of 80 cm of length and 0.28 mm ID and 0.61 mm OD from Smiths) was introduced 0.3 mm (approx.) in the ECA until the bifurcation of the CC. To avoid cell precipitation in the syringe, syringe was shaking during administration, that forced the use of manual injection instead of automatic pump. After cell administration, the catheter was removed and the arteriotomy site was ligated to prevent bleeding, the skin was closed, and the animal was allowed to recover from anesthesia.

### Intra-arterial administration dosage of MSCs

To determine the optimal cell dosage for i.a. cell administration, the effect of i.a. injection of two different cell doses on cerebral blood flow (CBF) was evaluated in healthy animals. Thus, 0.25 × 10^6^ and 1 × 10^6^ MSCs suspended in 300 μL PBS and vehicle were administered i.a. following the procedure indicated in case D (N = 6, each group). Following same protocol used for cell labeling characterization (see Supplemental Information), MSCs were incubated during 24 h with D-MNP, washed an incubated again during 12 h. Less than 15 min before animal injection, cells where detached and suspended in PBS.

To analyze the impact of cell administration on CBF, three laser Doppler probes were placed onto the squamosal bone in the skull to cover the territory of the MCA, and CFB was monitored prior and during the cell injection and for 1 h after administration. Twenty four hours after cell delivery, MR T_2_-weighted images were performed to evaluate the brain. Workflow of cell labeling, cell animal injection and MRI scanning is represented in Supplemental Figure S5.

The cell dose and therapeutic window were selected based on previous meta-analysis^39^.

### Biodistribution of MSCs after intra-arterial administration by means of MRI and histological analysis

One million CFSE-D-MNP labeled MSCs suspended in 300 μL PBS were administered i.a. following the procedure indicated in procedure D in healthy animals (N = 6). Four hours after cell delivery, MR T_2_*-weighted images were acquired to evaluate brain engraftment of the cells. Immediately after MRI, animals were sacrificed for immunohistological analysis and for transmission electronic microscopy (TEM). A scheme of cell labeling, cell animal injection and MRI scanning is represented is showed in Supplemental Figure S5.

### Biodistribution of MSCs after intravenous administration

Intravenous (i.v.) administration was performed through the jugular vein in healthy animals (N = 6). For that purpose, a 0.5 cm incision was made in the animal’s neck just above the clavicle and 1 cm to both the left and right of the midline. Subcutaneous fat was cut, the jugular vein was exposed, and 1 × 10^6^ CFSE-D-MNP labeled MSCs suspended in 300 μL PBS were administered manually using a 30gauge needle. An MRI was performed 4 h after administration to detect MSCs. Finally, the animals were sacrificed for further histological analysis immediately after the MRI. Workflow of cell labeling, cell animal injection and MRI scanning is represented in Supplemental Figure S5. The cell dose and therapeutic window were selected based on previous meta-analyses^39^.

### Neurorecovery study

To evaluate the effect of the administration route (i.a. *vs*. i.v.) on the neurorecovery efficacy of MSCs in stroke animal models, 4 groups were considered: Group 1 (N = 6): tMCAo treated with 300 μL PBS i.v. (jugular vein) 4 h after the onset of ischemia; Group 2 (N = 6): tMCAo treated with 300 μL 1 × 10^6^ MSCs labeled with D-MNPs i.v. (jugular vein) 4 h after the onset of ischemia; Group 3: tMCAo treated with 300 μL PBS i.a (procedure D) 4 h after the onset of ischemia; and Group 4 (N = 6): tMCAo treated with 300 μL 0.25 × 10^6^ D-MNP labeled MSCs and PBS i.a. (procedure D) 4 h after the onset of ischemia.

Animals were evaluated by means of infarct volume, functional deficit, blood cytokine levels, and histology analysis. Infarct volume sizes were measured using ADC maps, calculated from MR diffusion weighted imaging (DWI) acquired during occlusion (t = 0) to determine the basal ischemic damage before cell administration, and on T_2_-maps, obtained from T_2-_weighted images at days 1, 3, 7, and 14 after the induction of ischemia. Infarct volume sizes were expressed as percentages with respect to hemisphere volume corrected by edema factor. T_2_*-weighted images were acquired to track the injected cells and to evaluate hemorrhagic lesions 4 hours, 1, 3, 7, and 14 days after ischemia. In the Supplemental Figure S5 is showed all procedure for cell labeling, cell animal injection and MRI scanning. A cylinder test was used to evaluate the functional deficit and was performed prior to ischemia to assess the basal locomotor symmetry of the animals and on days 7 and 14 after ischemia. Venous blood samples (250 μL) were also collected during the occlusion 4 and 8 hours and, 1, 3, 7, and 14 days after ischemia to obtain further VEGF analysis. Finally, on day 14, animals were transcardially perfused and their brains processed for further histology analyses.

### Magnetic resonance imaging

MRI studies were conducted on a 9.4 T MR system (Bruker Biospin, Ettlingen, Germany) with 440 mT/m gradients. *In vitro* and *in vivo* magnetic resonance imaging is described in detail in Supplementary Data.

### Behavioral cylinder test

Forelimb contacts while rearing were scored with a total of 10 contacts recorded for each animal. The number of impaired and non-impaired forelimb contacts was calculated as the percentage of total contacts. The cylinder test was performed prior to tMCAo and 7 and 14 days after ischemia.

### Histological analysis

Histological analysis of cells location was also performed by means of TEM and immunohistology analysis. See Supplementary Data for information about histological protocols.

### VEGF analysis in serum samples

Venous blood was collected from the tail vein prior to MCAo, 4, 8 and 24 h, and 1, 3, 7, and 14 days after the onset of ischemia. Blood serum was obtained and stored at −80 °C until VEGF determination. ELISA assays were performed using a Quantikine ELISA Rat VEGF kit (R&D Systems, Inc., MI, USA) following the commercial indications. Detection limits were 31.2 pg/mL.

### Statistical analyses

All data were presented as the mean and standard deviation (mean ± SD). Two-way analysis of variance (ANOVA) followed by post-hoc Bonferroni evaluation was used for multiple groups to determine significant differences. Statistical significance was set at *P* < 0.05. Statistical analyses were conducted using IBM SPSS Statistics for Macintosh, version 18.0 (SPSS Inc., IL, USA).

## Additional Information

**How to cite this article**: Argibay, B. *et al*. Intraarterial route increases the risk of cerebral lesions after mesenchymal cell administration in animal model of ischemia. *Sci. Rep.* **7**, 40758; doi: 10.1038/srep40758 (2017).

**Publisher's note:** Springer Nature remains neutral with regard to jurisdictional claims in published maps and institutional affiliations.

## Supplementary Material

Supplementary Information
