# Supplementary material for: Intraarterial route increases the risk of cerebral lesions after mesenchymal cell administration in animal model of ischemia
Source: Sci Rep. 2017 Jan 16;7:40758. doi: 10.1038/srep40758 (PMC5238501; doi:10.1038/srep40758)
Supplement: Supplementary Information [file srep40758-s1.pdf]

SUPPLEMENTAL MATERIALS AND METHODS

Intraarterial route increases the risk of cerebral lesions after mesenchymal cell administration in animal model of ischemia.

Bárbara Argibay PhD[1], Jesse Trekker PhD[2,3], Uwe Himmelreich PhD[3], Andrés Beiras MD PhD[4], Antonio Topete PhD[5,6], Pablo Taboada PhD[5],María Pérez-Mato PhD[1], Alba Vieites-Prado BsC[1], Ramón Iglesias-Rey PhD[1], José Rivas PhD[7], Anna Planas PhD,[8] Tomas Sobrino PhD[1], José Castillo MD PhD[1*], and Francisco Campos PhD[1*]

[1] Clinical Neurosciences Research Laboratory, Clinical University Hospital, Health Research Institute of Santiago de Compostela (IDIS), Universidade de Santiago de Compostela. Santiago de Compostela, Spain.

[2]IMEC, Department of Life Science Technology, Leuven 3001, Belgium.

[3]Biomedical MRI, Department of Imaging and Pathology, KU Leuven, Leuven 3000, Belgium.

[4]Department of Morphological Sciences, Universidade de Santiago de Compostela. Santiago de Compostela, Spain.

[5]Grupo de Física de Coloides y Polímeros, Departamento de Física de la Materia Condensada, Universidade de Santiago de Compostela. Santiago de Compostela, Spain.

[6]Departamento de Fisiología, Centro Universitario de Ciencias de la Salud, Universidad de Guadalajara, Guadalajara 44340, México

[7]Applied Physics Department, Campus Vida, Universidade de Santiago de Compostela. Santiago de Compostela, Spain.

[8]Department of Brain Ischemia and Neurodegeneration, Institut d'Investigacions Biomèdiques de Barcelona (IIBB), Consejo Superior de Investigaciones Científicas (CSIC), Barcelona, Spain; August Pi i Sunyer Biomedical Research Institute (IDIBAPS), Barcelona,

SUPPLEMENTAL MATERIALS AND METHODS

Synthesis of dextran coated superparamagnetic nanoparticles

D-MNPs were prepared following a previously described protocol[1] with several modifications. Briefly, aqueous solutions of 0.1M Fe (III) (Sigma-Aldrich, St. Louis, MO, USA) and 0.1M Fe(II) (Sigma-Aldrich, St. Louis, MO, USA) in a 2:1 volume ratio were mixed (400 rpm) under N_2 atmosphere and T = 60 °C. Then, dextran (100 mg in 5 mL) from *Leuconostoc* spp. (Sigma-Aldrich, St. Louis, MO, USA) was added to the mixture. Ammonium hydroxide (5 mL; 5 mol/L) (Merck, Darmstadt, Germany) was added drop-wise to produce the particles. The solution was cooled to 23 ± 2°C and dialyzed (D0530, Sigma-Aldrich, St. Louis, MO, USA) against distilled water.

Characterization of D-MNPs

A structural analysis of the D-MNPs was performed by transmission electron microscopy (TEM) and X-ray diffraction (XRD). Samples were measured on a TEM microscope model Philips CM-12 operating at 120 kV with a Philips powder diffractometer fitted with Philips PW1710 control unit, vertical Philips PW1820/00 goniometer, and a FR590 EnrafNonius generator. Magnetic properties were recorded on a vibrating sample magnetometer (VSM) (Quantum Design TM, CA, USA). Iron content was determined by inductive coupled plasma optical emission spectroscopy (ICP-OES) (Varian Inc., Palo Alto, CA, USA), and the mean hydrodynamic particle size was measured using a dynamic light scattering (DLS) ALV-5000F instrument (ALV-GmbH, Germany) with vertically polarized incident light (θ = 488 nm) supplied by a diode-pumped Nd:YAG solid-state laser (Coherent Inc., CA, USA). Zeta-potentials were measured using a Zetasizer NanoZS instrument (Malvern, UK) equipped with a red laser (633 nm) in backscatter mode at 25 ºC. Polymer adsorption onto the cores was confirmed by Fourier-transform infrared (FTIR) spectroscopy with a FTIR spectrometer (FTIR 670, Varian Inc., Palo Alto, CA, USA) and quantified by thermal gravimetric analysis (TGA) on a Q5000IR (TA instruments, New Castle, DE, USA).

Cell lines

All experiments were performed using bone marrow rat MSCs purchased from Cultrex (Trevigen, Gaithersburg, MD, USA) and cultured in Iscove's Modified Dulbecco's Media (IMDM 78%), fetal bovine serum (10%), horse serum (10%), and penicillin-streptomycin (1%) (Gibco, Invitrogen, Paisley, UK), and Amphotericin-B (1%) (Sigma-Aldrich, St. Louis, MO, USA). Cell passage numbers between 7 and 18 were used in this study.

Cell labeling with D-MNPs combined with Poly-L-Lysine

MSCs were labeled following the protocol previously described.[2] Briefly, MSCs were incubated with 1.5 µg/mL of poly-L-lysine (PLL) combined with D-MNPs (100 µg/mL) for 24 h. Then, the D-MNP-containing medium was removed, and cells were washed 3 times with 1.5 mL of phosphate buffered saline (PBS) without Mg^{2+} and Ca^{2+} (Gibco, Invitrogen, Paisley, UK) to remove non-attached D-MNPs. After washing, cells were further incubated in fresh medium. After 12 h, the medium was removed and cells were washed with 1.5 mL of PBS and 0.5 mL of fresh medium. Finally, EDTA-trypsin (Gibco Invitrogen, Paisley, UK) was added to detach the cells from the well. Trypsin was neutralized with fresh medium, and the detached cells were collected.

Labeled and non-labeled MSCs were plated sequentially to evaluate the possible cellular toxicity and labeling clearance. Characterization of MSCs after labeling was performed different cellular points: 12 h (P0), 3 days (P1), and 5 days after D-MNP labeling (P2).

Characterization of D-MNP labeling of MSCs

Total cell counts were performed using Trypan Blue stain (STEMCELL Technologies, Grenoble, France) and a Neubauer counting chamber (Blaubrand, Sigma-Aldrich, St. Louis, MO, USA). Samples were diluted 1:5 with PBS and 1:2 with Trypan Blue. Cell counts were performed using an inverted microscope (Olympus IX51, Shinjuku, Tokyo, Japan).

Cell viability assays were conducted using the lactate dehydrogenase assay (LDH) kit (Sigma-Aldrich, St Louis, USA), following the manufacturer’s protocol. Lysed cell supernatants were also included (negative control). In brief, supernatants were centrifuged at 1000 rpm for 5 min

and further incubated with LDH-reagents for 20 min. Then, the plate was read in a Synergy2 plate reader (Biotek Instruments, VT, USA) at 490 nm, and the viability rate (%) was calculated with respect to control and lysate values.

Iron uptake was quantified by dissolving 1×10^5 cells in 1 mL HCl 37% extra pure (Merck, Darmstadt, Germany). Distilled water was added to achieve a total volume of 5 mL. Iron concentration was determined by inductive coupled plasma optical emission spectroscopy (ICP-OES) (Varian Inc., Palo Alto, CA, USA). The intensity of the emission line at 238.204 nm was measured for iron and compared to a standard solution.

Prussian Blue (PB) staining was performed to assess the presence of MNPs in the cells. Labeled and non-labeled cells from each time point were plated, washed with PBS after 8 h, and incubated for 20 min with a mix of equal parts of aqueous solutions of 20% HCl 37% extra pure and 10% potassium ferrocyanidetrihydrate (KFe(CN)6•3H2O, FW 422.2; Sigma-Aldrich, Paisley, UK). After incubation, cells were washed 3 times with PBS and images were taken using an inverted microscope (Olympus IX51, Shinjuku, Tokyo, Japan).

Phenotype characterization of MSCs after labeling was performed by flow cytometry. MSCs (0.25×10^6 cells) were washed with 50 µL PBS and stained by incubation (15 min) with 5 µL CD90-PerCP, 2.5 µL CD45-PE (BD Pharmigen, CA, USA), and 1 µL CD73-APC antibodies (Inmunostep, Salamanca, Spain). Then, 20,000 cell events were acquired in a BD FACS Aria I (BD Biosciences, CA, USA), and data were analyzed using FACSDiva software (BD Biosciences, CA, USA). MSCs were separated by their forward and side scattering signals and were characterized as CD90+CD73+/CD45- cells.

Angiogenic capacities were evaluated by tube formation analysis using Matrigel in labeled and non-labeled cells (BD Pharmigen, CA, USA). Angiogenic assay medium was prepared with 95% IMDM (Gibco. Invitrogen, Paysley, UK) and 5% FBS (Gibco, Invitrogen, Paisley, UK). Three hundred microliters of Matrigel (4 ºC) per well were added to a 24-well plate and incubated until solidification (30 min). Then, 80,000 labeled and non-labeled MSCs dispersed in angiogenic assay media were placed and well distributed in each well. After 6 h incubation,

images of tubular structures were taken using an inverted microscope (Olympus IX51, Shinjuku, Tokyo, Japan).

VEGF concentrations of labeled and non-labeled cell media were measured with a Quantikine VEGF ELISA kit (R&D Systems, MN, USA) following the manufacturer's protocol. VEGF values were corrected for the amount of total cell protein determined by Bradford techniques.[3] In brief, 400 µL 0.2% NaOH (sodium hydroxide pellets PA-ACS-ISO. Panreac, Barcelona, Spain) were added to each well, and the plate was placed under shaking for 4 h at 23 ± 2°C. Standard curves were generated from different concentrations of Bovine Serum Albumin (BSA) (cell culture grade, pH 7.0, lyophilized powder, GE Healthcare Life Sciences, PA, USA). Each sample (10 µL) was mixed with 190 µL distilled water and 50 µL Protein Assay Dye Reagent Concentrate (Bio-Rad, CA, USA) and the plate was read in the Synergy2 (Biotek Instruments, VT, USA) at 595 nm.

Magnetic resonance imaging

All studies were conducted on a 9.4T horizontal bore magnet (Bruker BioSpin, Ettlingen, Germany) with 440 mT/m gradients and a combination of a linear birdcage resonator (7 cm in diameter) for signal transmission and a 2 × 2 surface coil array for signal detection. A quadrature volume coil (7 cm in diameter) was also used in phantom studies.

MRI post-processing was performed using ImageJ software (W. Rasband, NIH, USA).

In vitro magnetic resonance imaging

Agar phantoms loaded with D-MNPs were made following a previously[2] with different Fe concentrations: 0.09, 0.05, and 0.02 mmol/L. T_2-weighted images were acquired using a multi-slice multi-spin-echo sequence (MSME) with 10.44 ms echo time, 3 s repetition time, 16 echoes with 10.4 ms echo spacing, 50 KHz spectral bandwidth, flip angle (FA)= 110º, 14 slices of 1 mm, 1 average, field of view (FOV) of 75 × 75 mm^2 (with saturation bands to suppress signal outside this FOV), a matrix size of 256 × 256 (in-plane resolution of 293 µm/pixel × 293 µm/pixel) and implemented without fat suppression option.

Agar phantoms of labeled and non-labeled cells were made to assess the detection capabilities of D-MNPs by magnetic resonance following a previously described procedure[2] with $1X10^5$ cells per condition. T_2*-weighted images were acquired using a multi gradient echo (MGE) sequence with 4.44 ms echo time, 1.8 s repetition time, 16 echoes with 6.75 ms echo spacing,100 KHz spectral bandwidth, FA= 30º, 14 slices of 1 mm, 2 averages, 75 × 75 mm^2 FOV (with saturation bands to suppress signal outside this FOV),a matrix size of 256 × 256 (in-plane resolution of 293 μm/pixel × 293 μm/pixel) and implemented with fat suppression option.

In vivo magnetic resonance imaging

Basal ischemic lesion during MCA occlusion was determined by counting pixels with apparent diffusion coefficient (ADC) values below a threshold in the ipsilateral brain hemisphere. The values of ADC in the healthy rat brain normally do not fall below 0.55 $x10^{-3}$ mm^2/s; therefore, this threshold provides a convenient means of segmenting abnormal tissue.[4] ADC maps were obtained from diffusion-weighted images (DWI) using a spin echo echo-planar imaging sequence (DTI-EPI) with the following acquisition parameters: 26.91 ms echo time, 4 s repetition time, 200 KHz spectral bandwidth, 7 b-values of 0, 300, 600, 900, 1200, 1600, and 2000 s/mm^2, FA= 90º, 4 averages, 14 consecutive slices of 1 mm, 24 × 16 mm^2 FOV (with saturation bands to suppress signal outside this FOV), a matrix size of 96 × 64 (isotropic in-plane resolution of 250 μm/pixel × 250 μm/pixel) and implemented with fat suppression option.

To evaluate the status of ACM occlusion in a noninvasive manner, the time-of-flight magnetic resonance angiography (TOF-MRA) was performed. The TOF-MRA scan was performed with a 3D-Flash sequence with a 2.5 ms echo time, 15 ms repetition time, FA= 20º, 2 averages, 98 KHz spectral bandwidth,1 slice of 14mm, 30.72 × 30.72 × 14 mm^3 FOV (with saturation bands to suppress signal outside this FOV), a matrix size of 256 × 256 × 58 (resolution of 120 μm/pixel × 120 μm/pixel × 241 μm/pixel) and implemented without fat suppression option.

DWIs were acquired during ACM occlusion, and TOF-MRA was performed during ACM occlusion and 4h after stem cell injection.

For MRI cell detection of labeled cells in the head of the animal, T_2*-weigthed images were acquired using a MGE sequence with a 2.9 ms echo time, 1.5 s repetition time, 16 echoes with 3.28 ms echo spacing,100 KHz spectral bandwidth, FA= 30º, 14 slices of 1 mm, 2 averages, 19.2 × 19.2 mm^2 FOV (with saturation bands to suppress signal outside this FOV), a matrix size of 192 × 192 (isotropic in-plane resolution of 100 μm/pixel × 100 μm/pixel) and implemented with fat suppression option.

The progression of ischemic lesions, infarct and edema volumes were determined from T2-maps calculated from T_2-weighted images. T_2-weighted images were acquired 24 h and 1, 3, 7 and 14 days after the onset of ischemia using a MSME sequence with a 9 ms echo time, 3 s repetition time, 16 echoes with 9 ms echo spacing, FA = 180º, 2 averages,75 KHz spectral bandwidth, 14 slices of 1 mm, 19.2 × 19.2 mm^2 FOV (with saturation bands to suppress signal outside this FOV), a matrix size of 192 × 192 (isotropic in-plane resolution of 100 μm/pixel × 100 μm/pixel) and implemented without fat suppression option. Edema was estimated by measuring the volumes of the affected (V_{Les}) and contralateral (V_c) hemispheres and using the formula: edema (%) = 100*(V_{Les}-V_c)/V_c.

Histological analysis

Transmission electronic microscopy study

Animals were sacrificed under sevofluorane anesthesia (8%), and 6 samples of brain cortex and striatum were taken immediately after death. Fixation and post-fixation were performed in 2% glutaraldehyde or 1% OsO_4 in sodium cacodylate buffer. Inclusion was performed in Spurr's epoxy resin. Semithin sections (0.5 μm) were stained with Toluidine blue and examined under an optical microscope to select brain regions for further TEM analysis, and ultrathin sections (100 nm) were stained with uranyl acetate and lead citrate.

Immunohistology

Animals were anesthetized and perfused transcardially with 100 mL PBS 0.1M pH 7.4 and 150 mL 4% formaldehyde (VWR Chemicals, Leuven, Belgium). Brain, lung and heart were

removed carefully from the skull and sectioned in 2 mm slices in a matrix, post fixed by immersion in 4% formaldehyde overnight, dehydrated, and embedded in 4% paraffin (VWR International a/s, Albertslund, Denmark).

For cell detection by histology, paraffin sections of 10 µm were deparaffinized in xylol and rehydrated in a graded alcohol series. Sections were washed with 1X PBS, heated to 99 ºC in citrate buffer (Dako, Barcelona, Spain) for 20 min, and washed again with 1X PBS. Slides were incubated first with rabbit polyclonal antibody to CD31 (Abcam, Cambridge, UK) (1:25), horse serum (15%), Triton X-100 (0.2%), and PBS overnight. Slides were then incubated at room temperature for 1 h with biotinylated horse anti-rabbit IgG antibody (Vector Laboratories, CA, USA) (1:200) and PBST. After 3 washes with PBST, slides were incubated with DyLight 594 streptavidin (Vector Laboratories, CA, USA) (1:500) and PBST. To stain the nuclei, 10 µL Hoechst (Invitrogen, Paisley, UK) in 60 mL 1X PBS was added to the slides and incubated for 15 min before washing with PBS. Slides were mounted with fluorescence mounting medium (Life Technologies, CA, USA). Images were taken with an Olympus IX51 microscope (Shinjuku, Tokyo, Japan).

For angiogenesis studies, slides were incubated first with rabbit polyclonal antibody to CD31 (Abcam, Cambridge, UK) (1:25), mouse monoclonal antibody to Ki-67 (Dako, Barcelona, Spain) (1:30), horse serum (15%), goat serum (15%), Triton X-100 (0.2%), and PBS overnight. Slides were incubated at room temperature for 1 h with biotinylated horse anti-mouse IgG antibody (Vector Laboratories, CA, USA) (1:200), DyLight 488 goat anti-rabbit IgG antibody (Vector Laboratories, CA, USA) (1:100), Triton (0.2%), and PBS. After 3 washes with PBST, slides were incubated with DyLight 594 streptavidin (Vector Laboratories, CA, USA) (1:500), Triton X-100 (0.2%), and PBS. Then, 10 µL Hoechst (Invitrogen, Paysley, UK) in 60 mL 1X PBS were added to the slides, incubated for 15 min, and washed with PBS. Sides were mounted with fluorescence mounting medium. For neurogenesis determination, slides were incubated first with rabbit polyclonal antibody to DCX (1:500), mouse monoclonal antibody to ki-67 (Dako, Barcelona, Spain) (1:30), horse serum (15%), goat serum (15%), Triton X-100 (0.2%), and PBS overnight. Slides were then incubated at room temperature for 1 h with biotinylated

horse anti-mouse IgG antibody (Vector Laboratories, CA, USA) (1:200), DyLight 488 goat anti-rabbit IgG antibody (Vector Laboratories, CA, USA) (1:100), Triton X-100 (0.2%), and PBS. After 3 washes with PBST, slides were incubated with DyLight 594 streptavidin (Vector Laboratories, CA, USA) (1:500), Triton X-100 (0.2%), and PBS. Then, 10 µL Hoechst (Invitrogen, Paysley, UK) in 60 mL 1X PBS were added to the slides, incubated for 15 min, and washed with PBS. Slides were mounted with fluorescence mounting medium. Images were taken using a Leica DMI 6000 B and processed using LAS AF V.1.0.0 software (Leica Microsystems, IL, USA).

REFERENCES

1. Pardoe, H., Chua-anusorn, W., St. Pierre, T.G. & Dobson, J. Structural and magnetic properties of nanoscale iron oxide particles synthesized in the presence of dextran or polyvinyl alcohol. *Journal of Magnetism and Magnetic Materials* **225**, 41-46 (2001).

2. Trekker, J., *et al.* Sensitive in vivo cell detection using size-optimized superparamagnetic nanoparticles. *Biomaterials* **35**, 1627-1635 (2014).

3. Bradford, M.M. A rapid and sensitive method for the quantitation of microgram quantities of protein utilizing the principle of protein-dye binding. *Anal Biochem* **72**, 248-254 (1976).

4. Reith, W., *et al.* Multislice diffusion mapping for 3-D evolution of cerebral ischemia in a rat stroke model. *Neurology* **45**, 172-177 (1995).

SUPPLEMENTAL FIGURES

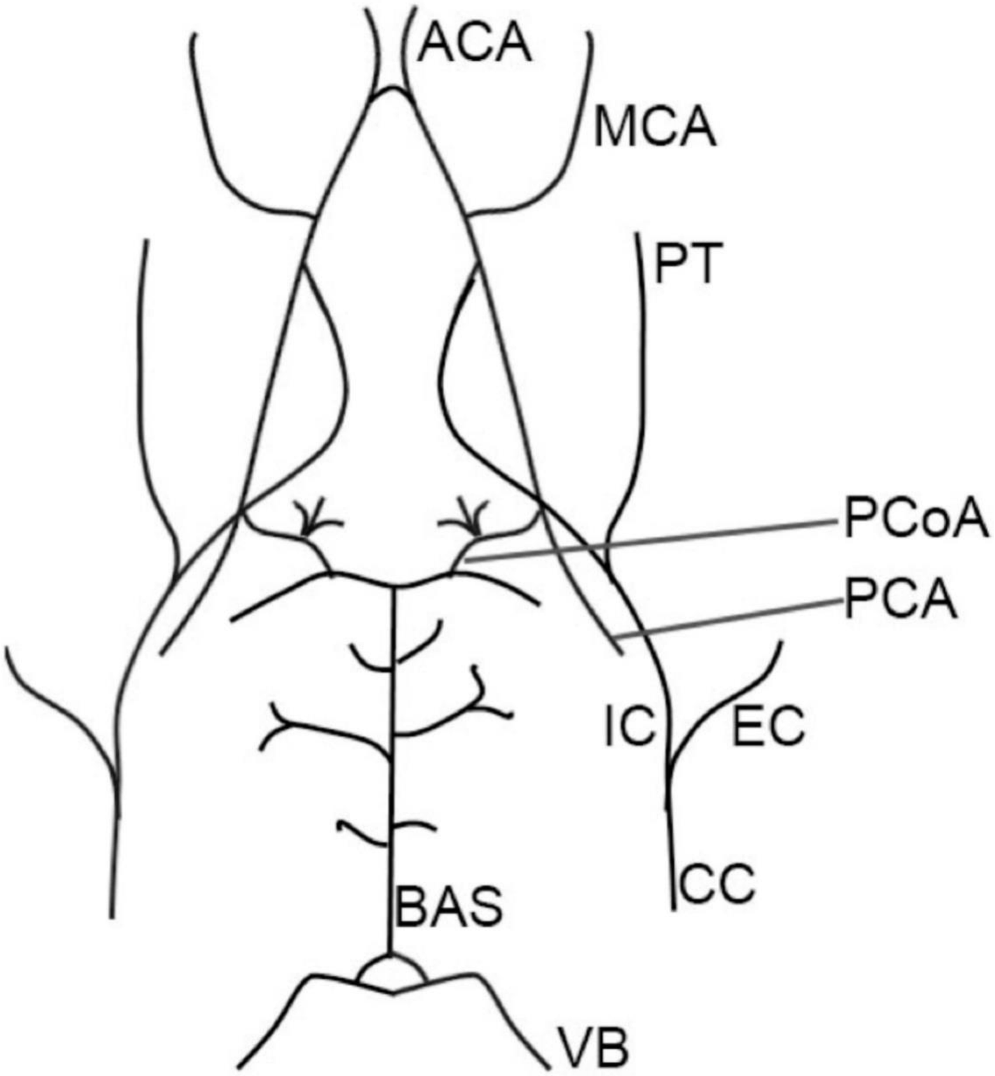

Supplementary Figure S1. Diagram of cerebrovascular anatomy of the rat. Since tMCAO is performed on the left side of the brain, the left side is considered the ipsilateral (Ip) side in this study. Abbreviations: Vertebral artery (VB), basilar artery (BAS), common carotid artery (CC), external carotid artery (EC), internal carotid artery (IC), pterigopalatin artery (PT), posterior cerebral artery (PCA), posterior communicating artery (PCoA), middle cerebral artery (MCA), and anterior cerebral artery (ACA).

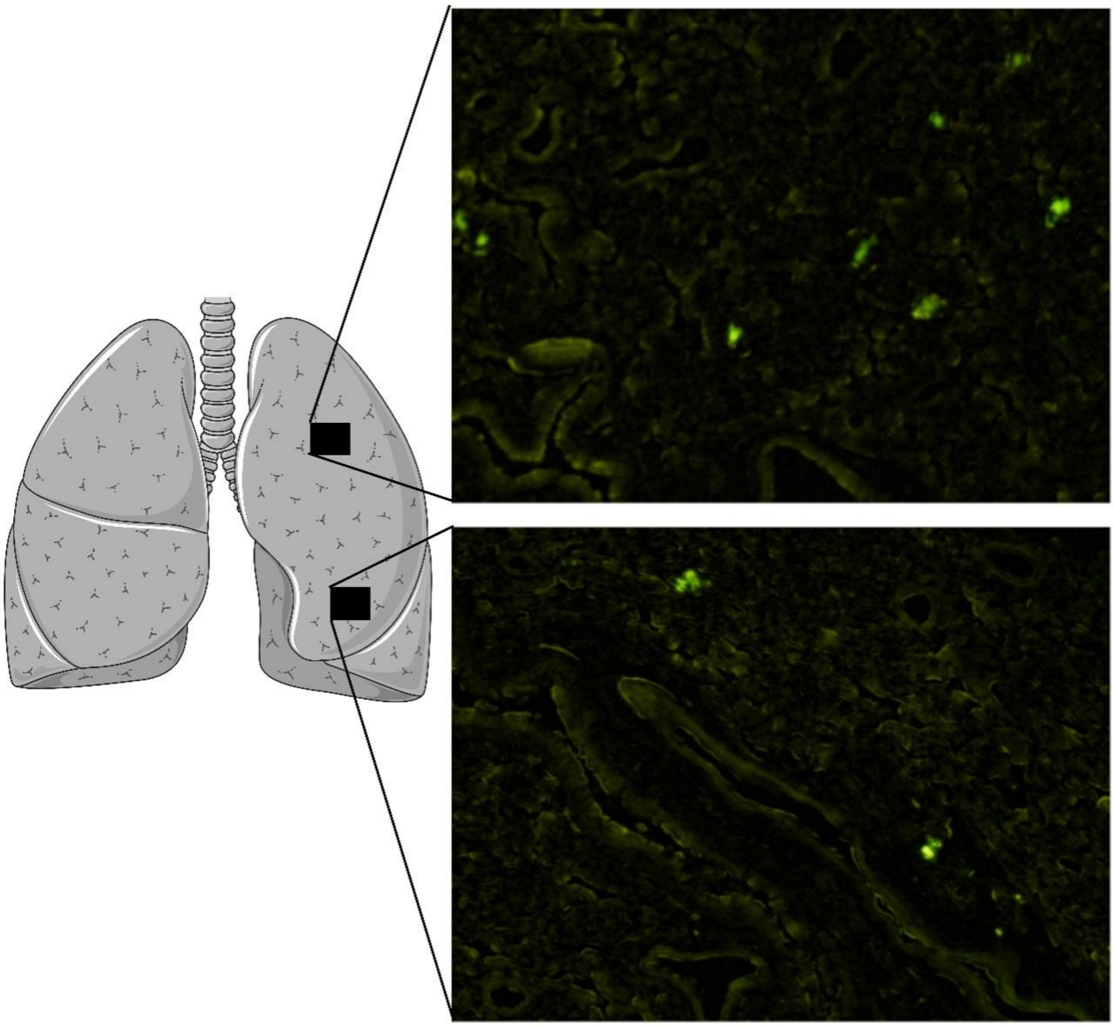

Supplementary Figure S2. Fluorescence optical microscopy of rat lung slices after i.v. administration of 1×10^6 D-MNP-CFSE-labeled MSCs. CFSE positive staining can be observed distributed along the lungs after jugular i.v. delivery. The figure was produced, in part, by using Servier Medical Art, (www.servier.com/Powerpoint-image-bank).

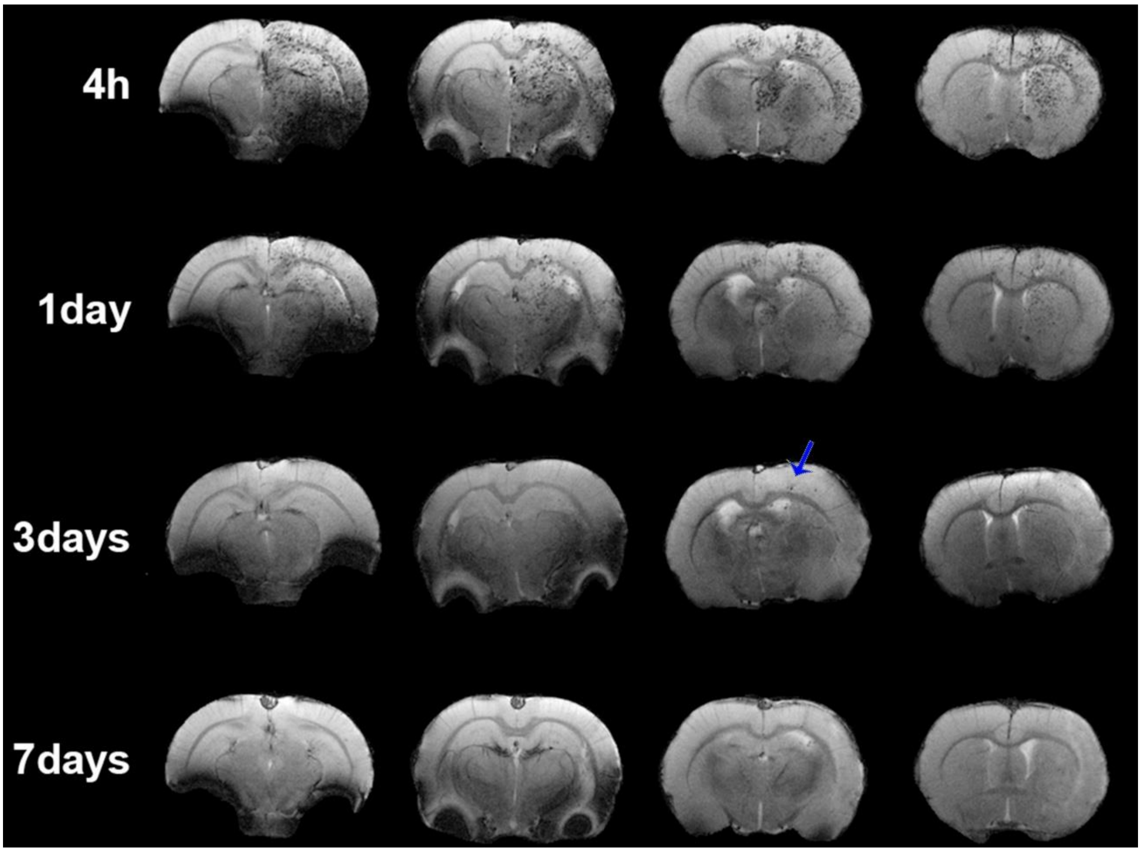

Supplementary Figure S3. *In vivo* cell tracking of D-MNPs labeled MSCs. MR T_2* weighted images of several brain slices of one ischemic animal injected with 0.25×10^6 D-MNP-labeled MSCs at 4 h and 1, 3, and 7 days after administration. Labeled MSCs are noted as black dots and can still be detected 3 days post administration (blue arrow).

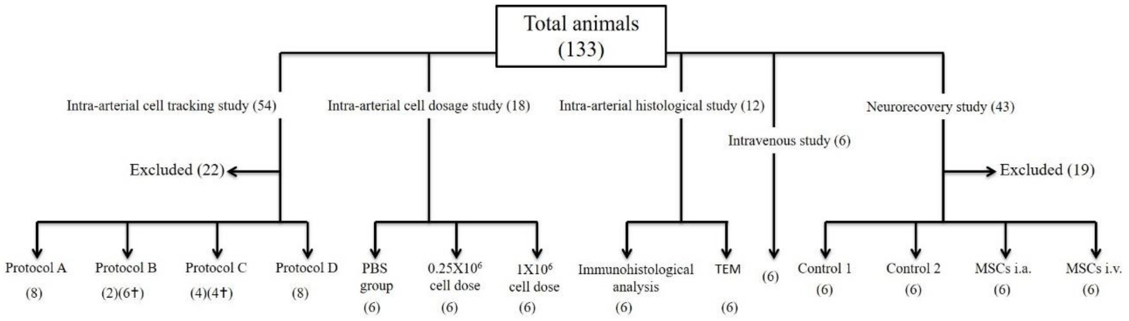

Supplementary Figure S4. Protocol diagram summarizing the number of animals included, with exclusion per group, for final analysis. Exclusion criteria were defined as described in Materials and Methods. (†) death animals. Excluded animals are defined as those animals which did not pass the inclusion criteria after ischemic surgery.

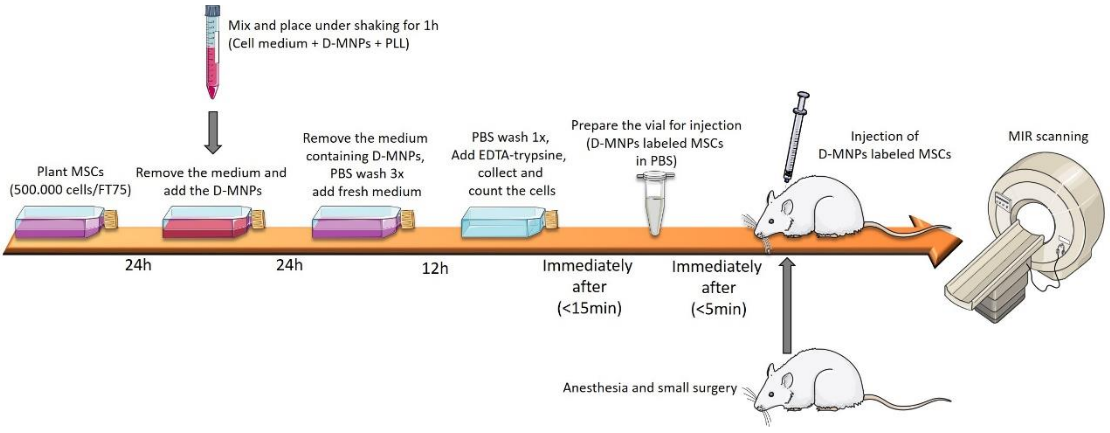

Supplementary Figure S5. Workflow of MSCs labeling with D-MNPs for *in vivo* administration: cell culture, cell D-MNPs labeling, cell animal administration and animal scanning in the MR. The figure was produced, in part, by using Servier Medical Art, (www.servier.com/Powerpoint-image-bank).

Conditions	Con-CC is closed during tMCAO	Ip-CC is open from MCA reperfusion until the injection	Ip-CC is open during the injection	Ip-CC remained opened after the injection
Case A	Yes	No	No	No
Case B	Yes	No	Yes	Yes
Case C	Yes	Yes	Yes	Yes
Case D	No	Yes	Yes	Yes

Supplementary Table S1. Common carotid configurations for animal models of tMCAO and intra-arterial (i.a.) delivery of labeled stem cells. Abbreviations: Contralateral common carotid (Con-CC) and Ipsilateral common carotid (Ip-CC).
